# Early COVID‐19 infection: The wide spectrum of extrapulmonary symptoms in elderly patients

**DOI:** 10.1002/agm2.12136

**Published:** 2020-11-29

**Authors:** Paolo Giovanardi, Barbara Manni, Antonella Vaccina, Lucia Cavazzuti, Chiara Salvia, Michela Latronico, Andrea Fabbo

**Affiliations:** ^1^ Primary Care Health Authority and Services of Modena Modena Italy; ^2^ Cardiology Unit ‐ Ospedale S.Agostino–Estense Azienda Ospedaliero‐Universitaria Modena Modena Italy; ^3^ Geriatric Service ‐ Cognitive Disorders and Dementia Primary Care, Health Authority and Services of Modena Modena Italy

Dear Editor,

we agree with the work of Rawle et al regarding the prevalence of novel Coronavirus‐19 (COVID‐19) atypical symptoms in institutionalized older adults. During the pandemic, we followed a geriatric cohort in a community hospital in Modena [mean age: 84.29 years (range: 75‐100 years), mean Cumulative Illness Rating Scale: 3.71 (range: 2‐6), mean Clinical Frailty Scale: 7 (range: 6‐8)]. The cohort had tested negative at COVID‐19 nasopharyngeal swab before hospital admission.

During hospitalization, two patients developed the common COVID‐19 flu syndrome as confirmed by the swab. Thus, the entire cohort was retested. Three asymptomatic patients had positive results, while three patients developed symptoms, as described below.

1. A 78‐year‐old female was admitted for rehabilitation after a femur fracture and was treated with Enoxaparin (4000 Units/d). The clinical framework changed with the appearance of episodes of hypotension along with swelling in the operated leg. The venous Doppler scan showed a deep vein thrombosis, and the blood count showed an increase of D‐dimer (18 773 ng/mL). The chest angio‐tomography revealed vascular defects in the right lung, suggesting a pulmonary embolization, and small areas of ground‐glass. The COVID‐19 swab tested positive.
2. An 85‐year‐old female with dilated cardiomyopathy and atrial fibrillation was treated with Apixaban. Diarrhea appeared without flu syndrome. The blood count revealed the increase of white blood cells (14 200/mm^3^) and of C‐reactive protein (8.9 mg/dL). Coproculture tested negative and the COVID‐19 swab tested positive.
3. An 89‐year‐old male suffering from osteoporosis, atrial fibrillation (treated with Dabigatran), and admitted for rehabilitation due to a femur fracture developed a hypokinetic delirium and a transient right hemiparesis. The brain tomography scan had negative results, the blood count revealed hypercalcemia (11.5 mg/dL), phosphoremia and serum albumin were reduced (2.3 mg/dL and 2.35 g/dL, respectively), parathormone was increased (102.4 pg/mL), and the COVID‐19 swab tested positive.

We observed the early stages of COVID‐19 disease in a geriatric cohort observing a wide spectrum of symptoms, ranging from asymptomatic subjects to the common flu and to acute cardiovascular and no‐ cardiovascular diseases. Patients developed typical and atypical symptoms simultaneously within 3 days, favored by the co‐occurrence with other diseases.

Uncommon COVID‐19 symptoms suggest the hypothesis of other ways of transmission, such as the oral‐fecal: this could explain the gastrointestinal symptoms and the viral detection in the gastrointestinal tract.^1^ Also, many neurologic symptoms have been described^2^ and COVID‐19 has the ability to cause venous and arterial thrombosis (even during anticoagulation) and induces the production of anticardiolipin antibodies.^3^

Our third case was difficult to interpret: the patient developed neurological symptoms and suffered from undiagnosed primary hyperparathyroidism. The association between COVID‐19 and hypercalcemia has not been reported but other coronaviruses (SARS, MERS, Deltacoronavirus) alter ion and especially calcium homeostasis.^4^, ^5^ The patient did not have symptoms attributable to hypercalcemia until COVID‐19 infection appeared. Conditions predisposing to hypercalcemia, such as primary hyperparathyroidism, may exaggerate the viral effects on ion homeostasis.

COVID‐19 is a multisystemic disease; atypical symptoms are common (even from the early stages and especially in geriatric patients) and have not been fully identified. During this pandemic great attention must be paid to all unexplained symptoms, especially in geriatric communities.

## CONFLICTS OF INTEREST

Nothing to disclose.

## AUTHOR CONTRIBUTIONS

Paolo Giovanardi, Barbara Manni, Antonella Vaccina, Andrea Fabbo: data collection, design, literature review, coordination, analysis, interpretation of data. All authors: writing of paper. All authors: critical revising.
